# Association between Metabolic Syndrome and the Number of Remaining Teeth in Postmenopausal Women: A Cross-Sectional Analysis Using the Korean National Health and Nutritional Examination Survey

**DOI:** 10.3390/jcm10204759

**Published:** 2021-10-17

**Authors:** Jeong-In Kim, Choong-Ho Choi, Ki-Ho Chung

**Affiliations:** 1 Department of Preventive & Public Health Dentistry, Chonnam National University, Gwangju 61186, Korea; kji6298@naver.com (J.-I.K.); hochoi@chonnam.ac.kr (C.-H.C.); 2 Dental Science Research Institute, School of Dentistry, Chonnam National University, Gwangju 61186, Korea

**Keywords:** tooth loss, metabolic syndrome, postmenopausal, oral health

## Abstract

There are very few studies on metabolic syndrome (MetS) and oral health in postmenopausal women. The purpose of this cross-sectional study was to determine the association between MetS and its components and the number of remaining teeth in postmenopausal women in Korea. The study selected 3320 menopausal women (40–79 years old) from those who participated in the seventh Korea National Health and Nutrition Survey (2016–2018). Multiple regression and multiple logistic regression analyses were performed to determine the association between MetS and its components and the number of remaining teeth. According to the multiple regression analysis, the regression coefficient (*B*) values were −1.62 (*p* < 0.05), −1.31 (*p* < 0.05), −1.60 (*p* < 0.05), and −2.28 (*p* < 0.05) in the hypertension group, hyperglycemia group, low high-density lipoprotein (HDL) cholesterol group, and MetS prevalence group, respectively. This indicates that the number of remaining teeth was lower in the MetS prevalence group as compared to the non-prevalence groups. As observed in the multiple logistic regression analysis, the odds ratio of the number of remaining teeth (less than 20) was higher in the MetS prevalence group (1.82 (*p* < 0.05)) as compared to the non-prevalence groups (1.25 (*p* < 0.05) in the abdominal obesity group, 1.50 (*p* < 0.05) in the hypertension group, 1.36 (*p* <0.05) in the hyperglycemia group, and 1.72 (*p* < 0.05) in the low HDL cholesterol group). Therefore, our results indicate that abdominal obesity, hypertension, diabetes, dyslipidemia, and prevalence of MetS are associated with tooth loss in postmenopausal women.

## 1. Introduction

In Korea, due to the rapid aging of the population, the number of people aged 65 and above accounted for 14.9% in 2019, and it is expected to increase to 25.0% in 2030 and 43.9% in 2060 [1]. Among women, the average age of menopause is 49.3 years [2], which comprises one-third of their lives. As a result, the importance of menopause in women is emerging in terms of life and health care. Menopause is defined as the permanent cessation of menstruation due to a decrease in ovarian hormone secretion, either spontaneously or by surgery, chemotherapy, or radiation [3,4]. The Korean Society of Menopause defines menopause as the absence of menstruation for a sustained period of more than 12 consecutive months after the last menstrual period. Regarding age at menopause, due to individual differences, menopause occurs at an average age of 48–52.

The prevalence of cardiovascular disease in women increases rapidly after the age of 40 years due to hormonal changes related to menopause, changes in the body due to aging, and increased fat accumulation [5]. Menopause is associated with diabetes, body mass index, and a family history of hypertension [6].

Oral health is an important part of menopausal women’s health as it can be the basis for improving the quality of life as well as maintaining and promoting health [7]. Teeth are essential organs for survival. As we age, salivation decreases, resulting in decreased taste and swallowing functions, and as the number of missing teeth increases due to an increase in the incidence of oral diseases, such as dental caries and periodontal disease, oral function and chewing ability deteriorate [8].

Risk factors for metabolic syndrome (MetS) include abdominal obesity, insulin resistance, excessive caloric intake, and insufficient physical activity. In addition, environmental, socioeconomic, and psychological factors are also known to influence MetS [9,10]. MetS is diagnosed when three or more of the following criteria are met: abdominal obesity, hypertension, hyperglycemia, hypertriglyceridemia, and low high-density lipoprotein (HDL) cholesterol levels. The prevalence of MetS globally has increased from 32.5% to 36.9% in the past 5 years [11], and in Korea, it has increased from 40.9% to 46.2% in the population aged 65 years and above [12]. MetS is a major risk factor for type 2 diabetes, coronary artery disease, and cerebrovascular disease. It affects the spread of these diseases [13] and is approximately three times more common than diabetes [14]; therefore, it is a syndrome that requires active management.

In addition, as the number of components of MetS increases, it has been reported that a relationship exists between periodontal disease and tooth loss [15], and recent studies have reported a relationship between MetS and tooth loss [16,17].

In a regional study on the relationship between MetS and periodontal disease in Korean menopausal and non-menopausal women, factors that significantly affected periodontal disease were non-use of dental floss, menopause, and an increase in the number of MetS factors [18]. In a global study on the association between MetS and periodontal disease measures in postmenopausal women, there was a significant difference in MetS and supragingival plaque, but no consistent association was found between the MetS components except for abdominal obesity and measures of periodontitis [19]. A study on the factors for tooth loss in postmenopausal women reported significant results according to the characteristics of osteoporosis and chronic diseases [20]. The studies on MetS and periodontal disease in postmenopausal women have been reported regionally and globally. However, there are still very few studies on MetS and the number of remaining teeth in postmenopausal women.

Therefore, this study aimed to provide basic data for promoting and maintaining the overall health and oral health of postmenopausal women by attempting to determine the relationship between MetS and its components and the number of remaining teeth in Korean postmenopausal women.

## 2. Materials and Methods

### 2.1. Study Design and Participants

This study used raw data from the seventh Korea National Health and Nutrition Survey (KNHANES) (2016–2018), which was conducted in accordance with the Declaration of Helsinki, and the protocol was approved by the Ethics Committee of the Institutional Review Board of the Korea Centers for Disease Control and Prevention (2018-01-03-P-A).

The purpose of this survey was to gather national data about the health status, health awareness and behaviors, as well as the nutritional intake of South Korean citizens. The sampling method of this survey included a complex, stratified, multistage, and probability-cluster survey of a large representative sample. The KNHANES consists of three items: a health survey, screening survey, and nutrition survey. In this study, the data were collected and analyzed using the health survey and oral examination survey.

The total number of participants in KNHANES (2016–2018) was 16,489 among 24,269 people who participated in oral examinations. In KNHANES, menopause was investigated by a self-reported method. Three postmenopausal women under the age of 40 were excluded because they were not representative cases, and all the participants over 80 years of age were also excluded because their age were recorded as 80 years instead of their actual age. Finally, 3320 postmenopausal women between the age of 40 and 79 were selected as participants for this study.

### 2.2. Assessment of MetS

MetS was defined using the National Cholesterol Education Program Adult Treatment Panel III and abdominal obesity standard of the Korean Society for the Study of Obesity, and it was diagnosed when three or more of the following criteria were met [21,22]: (1) abdominal obesity (waist circumference ≥ 85 cm for females); (2) high blood pressure (systolic ≥ 130 mmHg and diastolic ≥ 85 mmHg); (3) fasting serum glucose level ≥ 100 mg/dL; (4) hypertriglyceridemia (serum triglyceride level > 150 mg/dL); and (5) low high-density lipoprotein (HDL) cholesterol levels (<50 mg/dL for women).

### 2.3. Assessment of the Number of Remaining Teeth

Oral examinations were conducted by public health dentists affiliated with the Korea Centers for Disease Control and Prevention and public health dentists supported by cities and provinces.

For calculating the number of remaining teeth, a total of 32 teeth, including the third molars, were added. The remaining teeth were classified into two groups, “0–19” and “20 or more”, based on the 20 remaining teeth suitable for masticatory function [23].

### 2.4. Assessment of Confounders

In KNHANES (2016–2018), the items of education, economic activity, morbidity, and medical use of the health survey were surveyed by the interview method, and health behaviors such as smoking and drinking among the health survey items were surveyed in a self-reported format.

Among the variables investigated by the KNHANES (2016–2018), the sociodemographic characteristics and health status and behavior of the study participants, including the age, education level, household income, smoking status, drinking status, stress levels, oral examination history, daily toothbrushing frequency, use of oral hygiene products, and chewing and speaking challenges, were used as variables.

The educational level was classified as “≤elementary school”, “middle school”, “high school”, and “≥college”; and the household income level was classified as “lowest”, “second lowest”, “second highest”, and “highest”. The drinking status was classified as “no” for not drinking at all in the past year, “low” for drinking less than four times a month, and “high” for drinking more than 2–4 times a week. Smoking and occasional smoking were classified as “current”. Consequently, those who smoked in the past but were not currently smoking were classified as “past”, and those who never smoked were classified as “no”. Stress was classified as “very severe”, “severe”, “occasional”, and “rare” by using the usual level of stress perception in the form of a self-reported questionnaire in KNHANES. Dental visiting history within 1 year was classified as “yes” and “no”, if they had or had not undergone oral examinations in the past 1 year, respectively, and the daily brushing frequency was classified as “≤1”, “2”, and “≥3”. The use of oral hygiene products was classified as “yes” and “no”. Chewing and speaking problems were classified as “uncomfortable” for very uncomfortable and uncomfortable, and “not uncomfortable” for just so, not uncomfortable, and not at all uncomfortable, using the self-reported questionnaire format.

### 2.5. Statistical Analyses

Regarding the analysis of data, a complex sample design was applied, and the Statistical Package for the Social Sciences (SPSS), version 26.0 (IBM SPSS Statistics for Windows, Armonk, NY, USA), was used, and the statistical significance level was 0.05. A frequency analysis was performed to confirm the sociodemographic characteristics and health status and behavior, MetS of the study participants, and a *t*-test and one-way analysis of variance were performed to verify the difference in the number of remaining teeth according to the sociodemographic characteristics, health status, and behavior.

Multiple regression analysis and multiple logistic regression analysis were performed to verify the difference in the number of remaining teeth according to MetS and its components: abdominal obesity, hypertension, hyperglycemia, hypertriglyceridemia, and low HDL cholesterol. After adjusting for socio-demographic characteristics and health status and behavior, the difference between MetS and the number of remaining teeth was verified.

## 3. Results

### 3.1. The Number of Remaining Teeth According to Socio-Demographic Characteristics and Health Status and Behavior

Regarding the ages, 35.4% and 34.3% of the participants were in their 50s and 60s, respectively, and a high percentage of the participants (42.1%) had ≤ elementary school education. Drinking was highest at 60.0% in the low group, and the number of participants in smoking was highest at 93.1% in the no group. Regarding the frequency of daily brushing, 42% brushed two times and 49.1% brushed three times or more per day, and the rate of use of a proxabrush, dental floss, and a mouthwash was 86.0%, 82.3%, and 70.0% in the non-use group, respectively. Chewing problems and speaking problems were 31.1% and 11.7%, respectively, in the uncomfortable group (Table 1).

As age increased, the number of remaining teeth decreased, with the lowest number being 17.85 among those in their 70s. The number of remaining teeth was 19.82 for those with an education level ≤ elementary school and 19.30 for those in the lowest group of household income (*p* < 0.05). In addition, the group that did not receive oral examination last year had 21.92 remaining teeth, which was lower than that of the group that received (*p* < 0.05). Regarding dental floss and mouthwash use, the number of remaining teeth was 22.19 and 21.70 in the non-use group, respectively, which were lower compared to those in the use group (*p* < 0.05). The number of remaining teeth by chewing problems and speaking problems were found to be 20.04 and 15.88 in the uncomfortable group (*p* < 0.05). There was no statistically significant difference in the number of remaining teeth between the different categories of drinking status, smoking status, and proxabrush-use groups (*p* > 0.05, Table 1).

### 3.2. MetS Characteristics

Abdominal obesity, hypertension, hyperglycemia, hypertriglyceridemia, and low HDL cholesterol levels were observed in 35.9%, 38.6%, 43.1%, 27.7%, and 44.7% of the participants, respectively. The prevalence of MetS with three or more of these components was 33.2% (Table 2).

### 3.3. Association between MetS and the Number of Remaining Teeth

As a result of multiple regression analysis, the regression coefficient (*B)* values of the MetS component were −1.62 in the hypertension group, −1.31 in the hyperglycemia group, and −1.60 in the low HDL cholesterol group, indicating that the number of remaining teeth were lower than in the normal group (*p* < 0.05). The MetS prevalence group showed a lower *B* value (−2.28 (*p* < 0.05)), but there was no statistically significant difference after adjusting for the socio-demographic characteristics health status and behavior (*p* > 0.05, Table 3).

### 3.4. Association between MetS and the Number of Remaining Teeth Less Than 20

Among MetS components, the odds ratio of the number of remaining teeth less than 20 was 1.25 in the abdominal obesity group, 1.50 in the hypertension group, 1.36 in the hyperglycemia group, and 1.72 in the low HDL cholesterol group (*p* < 0.05). It was 1.82 in the MetS prevalence group (*p* < 0.05); however, there was no statistically significant difference after adjusting for sociodemographic characteristics and health status and behavior (*p* > 0.05, Table 4).

## 4. Discussion

The purpose of this cross-sectional study is to determine the relationship between MetS and its components and the remaining teeth in Korean postmenopausal women. Our study showed that MetS, abdominal obesity, hypertension, diabetes, and dyslipidemia are associated with tooth loss in postmenopausal women.

To preserve oral function, the World Health Organization (WHO) recommends retaining at least 20 teeth [24]. In this study, the average number of remaining teeth in the participants was 22.35; however, those in their 70s had 17.85 and did not meet the WHO recommendation. If the number of remaining teeth is low, the chewing ability, as well as the quality of life decreases [25,26]. Therefore, preserving the number of remaining teeth can greatly affect the quality of life and lifespan of the elderly population.

In this study, the number of remaining teeth decreased as the age increased and the education level and household income decreased, which was consistent with the findings of Shin [7]. The number of remaining teeth was significantly low in the group with less than one brushing frequency; in a study by Lee et al. [16], the number of remaining teeth was consistent with a low daily brushing frequency. The number of remaining teeth was significantly low at 15.88 (*p* < 0.05) for those who had difficulty in speaking, which is why tooth loss affects not only chewing function but also pronunciation and appearance [27], making it difficult to have a smooth social life, which suggests that an individual’s quality of life may decrease.

The multiple regression analysis revealed a significant difference in the number of remaining teeth in postmenopausal women in the hypertension, hyperglycemia, and low HDL cholesterol groups, and the MetS prevalence group showed a low number of remaining teeth (*p* < 0.05).

In addition, in the results of the multiple logistic regression analysis based on the number of 20 remaining teeth, there was a significant difference in the abdominal obesity, hypertension, hyperglycemia, and low HDL cholesterol groups; however, the MetS prevalence group showed a low number of remaining teeth (*p* < 0.05).

Kang’s [15] study showed that the odds ratio of missing teeth was significantly higher in the risk group than in the normal group in association with MetS risk factors and missing teeth, and that the risk of having less than 20 remaining teeth was high in the MetS prevalence group. This is consistent with the findings of a study by Lee [17], which showed significantly high values. In addition, in a retrospective study, 10.8% of patients with MetS lost at least one tooth over 5 years, and the risk of tooth loss increased by 1.54 times, similar to the findings of Furuta et al. [28]. Song and Lee [29] also reported that the number of remaining teeth was low in patients with chronic diseases, such as diabetes and hyperlipidemia. This was similar to the findings of a study by Oh [20], which showed a high prevalence of tooth loss in the hypertension, obesity, and disease morbidity groups, as well as a study by Kang et al. [30], which showed a 1.25- and 1.47-times higher loss of teeth in the hypertension and MetS groups, respectively, than in the non-disease group. In a study by Lee et al. [16], dyslipidemia and hypertriglyceridemia were significantly correlated with the number of remaining teeth, and the number of remaining teeth was low in people with MetS, similar to the findings of this study.

Several determinants, such as the age, education level, and demographic socioeconomic status, are associated with oral health-related behaviors, such as dental caries, periodontal conditions, and smoking in postmenopausal women, and tooth loss can be a complex measure of dental disease [31]. In addition, various chronic diseases are believed to cause inflammation in the teeth and periodontium and appear as a result of tooth loss [30]. In addition, postmenopausal hormone replacement therapy has a positive effect on maintaining natural teeth; therefore, the lack of hormone replacement therapy can be considered as an independent risk indicator for tooth loss in postmenopausal women [32]. In order to minimize risk factors for MetS in postmenopausal women and to preserve and manage remaining teeth, appropriate dental treatment, hormone therapy, dietary management, and exercise education are required.

This is a cross-sectional study that utilized data from the KNHANES to determine the association between MetS and the number of remaining teeth in Korean postmenopausal women, with the limitation of being unable to present an accurate causal association. In the case of self-reported questionnaires, the results may vary depending on recent memories. The investigation of the drug intake status of metabolic diseases in postmenopausal women was not conducted, which is another limitation of our study.

Future studies are needed to confirm the causality through longitudinal studies. However, using the KNHANES data, which possibly represent the national population, it was demonstrated that MetS is associated with the number of remaining teeth in postmenopausal women.

## 5. Conclusions

In postmenopausal women, MetS components were significantly associated with hypertension, hyperglycemia, and low HDL cholesterol levels in the multiple regression analysis. In the multiple logistic regression analysis, abdominal obesity, hypertension, hyperglycemia, and low HDL cholesterol levels were significantly correlated. As a result, we demonstrated that MetS may affect the number of remaining teeth in postmenopausal women.

## Figures and Tables

**Table 1:** Number of remaining teeth according to sociodemographic characteristics and health status and behavior.

Variables	Classification	*n*	%	M	SE	*p*-Value ^1^
Age	40–49	97	3.2	27.24	0.46	0.000
	50–59	1106	35.4	26.20	0.13	
	60–69	1170	34.3	22.78	0.27	
	70–79	947	27.1	17.85	0.41	
Education level	≤Elementary school	1474	42.1	19.82	0.31	0.000
	Middle school	571	17.3	23.47	0.31	
	High school	830	26.7	25.16	0.22	
	≥College	444	13.9	26.48	0.18	
Household income	Lowest	1017	29.0	19.30	0.38	0.000
	Second lowest	912	27.4	22.89	0.31	
	Second highest	719	22.3	24.55	0.23	
	Highest	663	21.3	25.61	0.21	
Drinking history	No	754	30.3	23.09	0.33	0.168
	Low	1478	60.0	23.76	0.21	
	High	229	9.7	22.83	0.79	
Smoking history	No	3095	93.1	22.91	0.17	0.077
	Past	104	3.0	21.82	0.99	
	Current	121	3.9	20.97	0.98	
Stress level	Very severe	176	5.6	23.24	0.59	0.001
	Severe	647	19.6	22.47	0.40	
	Occasional	1771	54.3	23.30	0.21	
	Rare	715	20.5	21.67	0.36	
Dental visiting history within 1 year	No	2237	67.4	21.92	0.22	0.000
	Yes	1083	32.6	24.61	0.21	
Daily toothbrushing frequency	≤1	315	8.9	17.76	0.66	0.000
	2	1412	42.1	22.40	0.26	
	≥3	1593	49.1	24.05	0.21	
The use of a proxabrush	Non-use	2848	86.0	22.71	0.19	0.099
	Use	472	14.0	23.36	0.37	
The use of dental floss	Non-use	2749	82.3	22.19	0.20	0.000
	Use	571	17.7	25.61	0.22	
The use of a mouthwash	Non-use	2379	70.0	21.70	0.23	0.000
	Use	941	30.0	25.36	0.16	
Chewing difficulty	Uncomfortable	1100	31.1	20.04	0.33	0.000
	Not uncomfortable	2207	68.9	24.05	0.17	
Speaking difficulty	Uncomfortable	424	11.7	15.88	0.54	0.000
	Not uncomfortable	2883	88.3	23.72	0.16	

M: mean; SE: standard error. ^1^ *p* < 0.05 using the one-way analysis of variance or *t*-test.

**Table 2:** Distribution of MetS and its components.

Variables	Classification	*n*	%
Abdominal obesity	<85 cm	2092	64.1
	≥85 cm	1219	35.9
Hypertension	<130/85 mmHg	1985	31.4
	≥130/85 mmHg	1333	38.6
Hyperglycemia	<100 mg/dl	1803	56.9
	≥100 mg/dl	138	43.1
Hypertriglyceridemia	<150 mg/dl	2295	72.3
	≥150 mg/dl	897	27.7
Low HDL cholesterol level	≥50 mg/dl	1728	55.3
	<50 mg/dl	1461	44.7
MetS ^1^	Less than 3	2086	66.8
	3 or more	1094	33.2

Using the frequency analysis. ^1^ MetS is defined when 3 or more of the following criteria are present: abdominal obesity, hypertension, hyperglycemia, hypertriglyceridemia, and low HDL cholesterol level. MetS, metabolic syndrome; HDL, high-density lipoprotein.

**Table 3:** The association between MetS and the number of remaining teeth.

Variables	Classification	B ^2^	SE	*p* ^1^	B ^3^	SE	*p* ^1^
Abdominal obesity	No	0.00					
	Yes	−0.65	0.37	0.076			
Hypertension	No	0.00					
	Yes	−1.62	0.38	0.000			
Hyperglycemia	No	0.00					
	Yes	−1.31	0.35	0.000			
Hypertriglyceridemia	No	0.00					
	Yes	−0.07	0.39	0.864			
Low HDL cholesterol level	No	0.00					
	Yes	−1.60	0.37	0.000			
MetS	No	0.00					
	Yes	−2.28	0.35	0.000	−0.13	0.34	0.711

SE: standard error. ^1^ *p* < 0.05 using the complex samples multiple regression analysis. ^2^ The crude regression coefficient. ^3^ Adjusted for the age, education level, household income, drinking status, smoking status, dental visiting history within 1-year, daily toothbrushing frequency, use of a proxabrush, use of dental floss, use of a mouthwash, chewing difficulty, and speaking difficulty.

**Table 4:** The association between MetS and the number of remaining teeth less than 20.

Variables	Classification	Crude OR (95% CI)	*p* ^1^	Adj OR ^2^ (95% CI)	*p* ^1^
Abdominal obesity	No	1.00			
	Yes	1.25 (1.01~1.54)	0.039		
Hypertension	No	1.00			
	Yes	1.50 (1.22~1.84)	0.000		
Hyperglycemia	No	1.00			
	Yes	1.36 (1.11~1.67)	0.003		
Hypertriglyceridemia	No	1.00			
	Yes	0.95 (0.76~1.20)	0.670		
Low HDL-cholesterol level	No	1.00			
	Yes	1.72 (1.38~2.15)	0.000		
MetS	No	1.00		1.00	
	Yes	1.82 (1.49~2.22)	0.000	1.09 (0.82~1.44)	0.570

Adj: adjusted; OR: odds ratio; CI: confidence interval. ^1^ *p* < 0.05 using the complex samples logistic regression analysis. ^2^ Adjusted for the age, education level, household income, drinking status, smoking status, dental visiting history within 1-year, daily toothbrushing frequency, use of a proxabrush, use of dental floss, use of a mouthwash, chewing difficulty, and speaking difficulty.

## Data Availability

The dataset analyzed for this study can be found at https://knhanes.kdca.go.kr/knhanes/eng/index.do (accessed on 17 October 2021).
